# Early Childhood Father Absence and Depressive Symptoms in Adolescent Girls from a UK Cohort: The Mediating Role of Early Menarche

**DOI:** 10.1007/s10802-014-9960-z

**Published:** 2014-11-20

**Authors:** Iryna Culpin, Jon Heron, Ricardo Araya, Carol Joinson

**Affiliations:** School of Social and Community Medicine, University of Bristol, Oakfield House, Oakfield Grove, Clifton, BS82BN UK

**Keywords:** Avon Longitudinal Study of Parents and Children (ALSPAC), Cohort study, Depressive symptoms, Father absence, Timing of menarche

## Abstract

Absence of the biological father in early childhood has been linked to depressive symptoms in mid-adolescent girls. Earlier studies have linked father absence to early timing of menarche, and early menarche is a risk factor for increased depressive symptoms in adolescence. No studies, however, have examined whether the association between father absence and depressive symptoms may be explained by the early onset of menarche. This study investigated whether age at menarche mediates the association between father absence in early childhood (birth to 5 years) and depressive symptoms in adolescent girls aged 14 years. The study sample comprised 7056 girls from a large UK birth cohort (Avon Longitudinal Study of Parents and Children) who provided data on age at onset of menarche and depressive symptoms assessed using the Short Mood and Feelings Questionnaire at 14 years. Mothers provided data on father absence from the birth of the study child up to 10 years. Using structural equation modelling, we found that 15 % of the total estimated association between father absence in early childhood and depressive symptoms at 14 years was explained by early age at menarche. In addition to the mediated effect, father absence was linked to an 11 % increase in depressive symptoms in adolescence. The findings suggest that early age at menarche is one of the pathways linking early childhood father absence and depressive symptoms in mid-adolescent girls. Preventive strategies could be targeting young girls at risk for depressive symptoms as a function of stressful family factors (e.g., biological father absence) and earlier menarche.

**Electronic supplementary material:**

The online version of this article (doi:10.1007/s10802-014-9960-z) contains supplementary material, which is available to authorized users.

Research on the role of fathers and fathering on child and adolescent psychological well-being has emphasised the complexity of the association between presence or absence of the biological father and mental health outcomes. The father-child relationship is characterised by an independent man-to-child attachment (Mackey 2001), which shapes development and is associated with both positive and negative mental health outcomes. However, compared with studies of maternal influence, there is a relative lack of research examining the impact of fathering on child and adolescent mental health (Phares 1992). Existing longitudinal evidence suggests that childhood family structure, including absence of the biological father, is a risk factor for development of depressive symptoms in adolescence (Cuffe et al. 2005; Culpin et al. 2013; Jaffee et al. 2002). Although yet inconclusive, a growing body of research suggests girls’ specific psychological vulnerability to family dissolution and father absence when compared to boys (Culpin et al. 2013; Oldehinkel et al. 2008; Storksen et al. 2006). Consistent with epidemiological research emphasising the critical role of early-life stress (e.g., actual or perceived loss of a parent) on subsequent development of psychopathology (Agid et al. 2000), father absence in early childhood (birth to 5 years) appears to exert a more pronounced negative effect on adolescent mental health (Culpin et al. 2013; Donahue et al. 2010; Lansford et al. 2001). There are, however, few studies that examine the mechanisms linking early father absence to increased risk of adolescent depression. Research into the mechanisms through which adverse childhood experiences, including absence of the biological father, influence adjustment is important for understanding the aetiology of adolescent depression.

It has been suggested that biological and psychosocial effects of advancing puberty may be implicated. In particular, early timing of puberty may be a potential mechanism that explains the association between early childhood father absence and development of depressive symptoms in adolescence. Life-course adversity theories such as psychosocial acceleration theory (Belsky et al. 1991) and paternal investment theory (Draper and Harpending 1982, 1988) emphasise the importance of certain developmental experiences and psychosocial cues in the early rearing environment that steer girls toward earlier versus later onset of sexual activity and reproduction. One such factor is father absence during the first 5 years of child’s life. Psychosocial acceleration theory (Belsky et al. 1991) hypothesises biological father absence as one of a range of stressors (including socioeconomic disadvantage, parental conflict, harsh and negative parenting) that influence timing of puberty, whilst paternal investment theory (Draper and Harpending 1982, 1988) stresses a unique and central role of fathers in shaping girl’s future pubertal and reproductive strategies, separate from the effects of other aspects of psychosocial stress and supports in child’s family environment. Specifically, psychosocial acceleration theory postulates that children whose early family experiences are high in stress, including that associated with biological father absence, are more likely to develop insecure attachments, experience earlier pubertal maturation and accelerated sexual activity than children whose early childhood environment is characterised by nurturing experiences (Belsky et al. 1991). Although both theories place different emphasis on the role of fathers influencing the timing of pubertal maturation in girls, they converge on the assumption that stressful early childhood experiences, including father absence, affect the physiological and hormonal mechanisms that initiate and control pubertal development (Ellis 2004). The accelerating effect of father absence on subsequent timing of menarche has been consistently reported across a range of cross-sectional (Bogaert 2008; Romans et al. 2003; Quinlan 2003) and longitudinal studies (Belsky et al. 2007; Culpin et al. 2014; Ellis and Garber 2000). In addition, there is evidence to suggest that exposure to father absence during the first 5 years of life is more strongly associated with early menarche than father absence later in childhood (Alvergne et al. 2008; Culpin et al. 2014; Moffitt et al. 1992).

Adolescence is the period associated with a rapid rise in rates of depression in females compared with males (Angold et al. 2002; Hankin et al. 1998). Given the female increase in depression coincides with puberty, aspects of pubertal development may explain this rise. The transition into puberty is a critical developmental period associated with profound biological and psychosocial changes, which place girls at increased risk of depressive symptoms (Stice et al. 2001). Numerous prospective studies have indicated that girls who experience menarche earlier than their peers have higher levels of depressive symptoms in adolescence (Kaltiala-Heino et al. 2003; Joinson et al. 2013, 2011). Although studies have found evidence linking father absence to early menarche and early menarche to increased risk of depression, to our knowledge, no studies have examined whether early menarche mediates the association between early childhood father absence and depressive symptoms in adolescence.

Father absence occurs in the context of multiple socioeconomic and familial adversities (e.g., financial hardship, maternal depression) that precede family dissolution and are associated with both earlier menarche (Ellis and Garber 2000; Ellis and Essex 2007) and adolescent psychopathology (Grant et al. 2006). It has been previously argued that the possibility of confounding by a variable common to both the mediator and the outcome may bias the estimation of mediation leading to inaccurate conclusions regarding the explanatory pathways (Robins and Greenland 1992). Thus, it is important to control for these factors whilst examining the pathways between early childhood father absence, age at menarche and depressive symptoms.

The current study, based on girls from a large contemporary UK cohort, examines the mediating effect of early menarche in the association between absence of the biological father during early childhood (birth to 5 years) and depressive symptoms at 14 years. We estimate the magnitude of the mediating effect whilst controlling for the effects of confounding variables related to various aspects of socioeconomic adversity and maternal characteristics.

## Method

### Participants

The sample comprised participants from the Avon Longitudinal Study of Parents and Children (ALSPAC: www.alspac.bris.ac.uk; Fraser et al. 2013). The ALSPAC is an on-going population-based study investigating a wide range of influences on the health and development of children. Ethical approval for the study was obtained from the local Research Ethics Committees and the study is monitored by the ALSPAC Law and Ethics Committee. Pregnant women resident in the former Avon Health Authority in south-west England, having an estimated date of delivery between April 1, 1991, and December 31, 1992 were invited to take part, resulting in a cohort of 14,541 pregnancies and 13,988 children (including 6762 girls) alive at 12 months of age. When the oldest children were aged 7 years, an attempt was made to increase the size of the initial sample with eligible cases that did not join the cohort at the outset. The phases of enrolment are described in more detail in the cohort profile paper (Boyd et al. 2013). The number of active new cases that are represented in the data source is 542 (294 females). Thus, the starting sample for the current study was 7056 girls. Of these individuals, 3073 girls provided data on depressive symptoms at 14 years, 5295 had data on father absence, and 4148 girls provided data on age at menarche. Incorporation of these data reduced the sample to 2675, with further reduction to 2057 girls with complete data on all confounders. Comparison of socio-demographic indicators across different samples is reported in Table 1.

**Table 1:** Distribution of socioeconomic, maternal and familial indicators in the original ALSPAC cohort of girls and in various sub-samples used in the study

Risk factor	Total sample of females in ALSPAC (*n* = 7056)	Sample with complete data on age at menarche onset (*n* = 4148)	Sample with complete responses on SMFQ at 14 years (*n* = 3073)	Full sample: complete data on age at menarche + complete SMFQ responses at 14 years + data on father presence/absence 0–10 years (*n* = 2675)	Complete sample: complete data on age at menarche + complete SMFQ responses at 14 years + data on father presence/absence 0–10 years + data on antenatal confounders (*n* = 2057)
	(%)	(%)	(%)	(%)	(%)
Social group					
Non-manual	50.8	55.7	57.4	58.6	58.6
Manual	49.2	44.3	42.6	41.4	41.4
Sample n	5618	3600	2714	2455	2057
Homeownership status					
Owned	73.4	81.3	83.4	84.2	85.8
Privately rented	26.6	18.7	16.6	15.8	14.2
Sample n	6344	3793	2818	2527	2057
Car access					
Yes	89.3	93.8	94.9	95.3	95.8
No	10.7	6.2	5.1	4.7	4.2
Sample n	6344	3786	2812	2521	2057
Major financial problems					
No	86.3	88.9	89.1	89.2	89.1
Yes	13.7	11.1	10.9	10.8	10.9
Sample n	5790	5183	2627	2365	2057
Mother’s educational qualifications					
≥High school	70.4	76.8	79.9	81.6	83.0
No qualifications	29.6	23.2	20.1	18.4	17.0
Sample n	6031	3775	2817	2541	2057
Family size					
<3	94.1	95.6	95.8	96.0	96.2
3+	5.9	4.4	4.2	4.0	3.8
Sample n	6306	3782	2809	2517	2057
Early parenthood					
No	95.2	97.7	98.3	98.5	99.0
Yes	4.8	2.3	1.7	1.5	1.0
Sample n	6756	3936	2906	2586	2057

*ALSPAC* Avon Longitudinal Study of Parents and Children; *SMFQ* Short Mood and Feelings Questionnaire

### Measures

#### Depressive Symptoms

The Short Mood and Feelings Questionnaire (Angold et al. 1995) is a brief (13-item) questionnaire for depressive symptoms enquiring about the occurrence of depressive symptoms over the past 2 weeks. Study children completed the SMFQ at research clinic at a mean age of 13 years 10 months (hereafter referred to as 14 years). The SMFQ correlates highly with more extensive depression rating scales such as the Children’s Depression Inventory (Kovacs 1992) and the Diagnostic Interview Schedule for Children (Shaffer et al. 2000). It has high internal reliability (Cronbach’s alpha = 0.90; Costello et al. 1991), discriminates depressed from non-depressed children in general population samples (Angold et al. 1995), and the internal construct validity of a single continuum of severity of depressive symptoms has been supported in a UK community sample in which the items were subjected to uni-dimensional item response modelling after simple binary recoding (Sharp et al. 2006).

#### Absence of the Biological Father

Because there is evidence to suggest stronger effect of early father absence on development of depressive symptoms in adolescence (Allison and Furstenberg 1989; Culpin et al. 2013; Ermisch and Francesconi 2001), we specifically focused on the period comprising father absence from birth to 5 years. Absence/presence of the biological father during this period was derived from the questionnaires given to study mothers at regular intervals since the birth of the study child (1 year 7 months, 2 years 7 months, 3 years 9 months, 7 years, 8 years, and 10 years) asking whether the present live-in-father-figure is the natural father of the study child, and, if not, how old the study child was when the natural father stopped living with the family (father present = 81.9 %; father absent = 8.2 %). Father absence was, therefore, defined as biological father absence from the household due to family breakdown.

#### Timing of Menarche

Menarche is a commonly used marker of the timing of female puberty (Parent et al. 2003). A series of nine postal questionnaires pertaining to pubertal development were administered approximately annually to the study mother from the time their daughter was 8 years old up to the age of 17 years. The questionnaire asked whether the menstrual periods had begun, and if so at what age (recorded in years & months). The first-reported age at onset was used, because these data ought to be the most accurate and least affected by recall bias (Koo and Rohan 1997). The mean age at onset of menarche was 12 years 6 months (SD = 1.17, range = 7.6–16.9 years), corresponding closely to that reported in other large contemporary studies based on samples from the US and Western European countries (Parent et al. 2003). Age at menarche was analysed as a continuous variable.

#### Confounding Factors

Increasing evidence indicates that failure to control for the mediator-outcome confounding may result in biased estimates of the direct and indirect effects, even when control for the mediator and confounders in the exposure–outcome regression is introduced (Cole and Hernan 2002; Pearl 2012; Robins and Greenland 1992). To address this concern, analyses were adjusted for socioeconomic disadvantage (e.g., financial problems, maternal education) and maternal characteristics (maternal depression, maternal age at menarche) that have been linked to earlier menarche (Ellis and Garber 2000; James-Todd et al. 2010) and are also associated with depression in adolescence (Gilman et al. 2003; Petterson and Albers 2001). In addition, risk of parental breakdown is higher in families with lower levels of household income and educational attainment (Hankin et al. 2010), higher levels of maternal depression (Agid et al. 1999), and for those living in rented accommodation (Lupien et al. 2009). Thus, the model was adjusted for maternal characteristics and indices of socioeconomic disadvantage derived from the antenatal questionnaires to estimate the effects of early childhood father absence whilst controlling for these variables.

Confounders were collected prospectively during the antenatal period. Indicators of socioeconomic disadvantage were: home ownership (owned accommodation versus privately rented); major financial problems (occurrence of major financial problems since pregnancy versus none); and maternal education, classified as the lowest (certificate of secondary school education or vocational qualification) and the highest (advanced-level qualifications generally obtained at age 18 years/university degree) levels of educational attainment. Maternal characteristics included: mothers’ age at menarche measured retrospectively at 12 weeks gestation (early: 8–11 years versus normative/late: 12–15 years); and maternal depression assessed at 18 and 32 weeks gestation using the Edinburgh Postnatal Depression Scale (EPDS; Cox et al. 1987) dichotomized at a cut-off of 12/13, the standard cut-off used to indicate probable depressive disorder (Evans et al. 2001).

#### Analytic Strategy

We examined whether missing data could have introduced bias to our results by comparing characteristics of missing children with the complete sample using chi-square test. Mediation analysis was performed using structural equation modeling (SEM) in Mplus version 7 (Muthén and Muthén 2012). We applied confirmatory factor analysis (CFA) to derive a normally distributed latent trait underlying the observed SMFQ scores using individual response items. Although a categorical approach is valuable for clinical practice and application, it has been argued that depressive symptoms may be better represented as a continuum (Hankin et al. 2005) and that a dimensional approach is preferred for hypothesis testing (Kraemer et al. 2004). Previous studies in ALSPAC have examined depressive symptom latent traits derived from the SMFQ (Joinson et al. 2013, 2011) to address the limitations associated with categorisation of continuous varaibles. We used mediation approach recommended by Muthén (Muthén 2011) to assess mediation effects within the context of mediator-outcome confounding. The ‘Model Constraint’ command was utilized to derive new parameters and standard errors representing causally-defined direct and indirect effects (Robins and Greenland 1992) from model estimated parameters. The dependent variables in the mediation model were depressive symptoms and age at menarche which were allowed to co-vary with each other. All confounders in the model were binary indicators. The key focus was a binary risk factor defined as father presence versus absence from birth to 5 years. Analyses were adjusted for indices of socioeconomic disadvantage (financial problems, homeownership status, maternal education) and maternal characteristics (antenatal depression and age at menarche).

#### Missing Data

Sample attrition led to a substantial amount of missing data, particularly for the outcome and the confounders (20–25 %). We used multiple imputation by chained equations (MICE) to impute missing data on the outcome and confounders (White et al. 2011) using the *ice* command in Stata version 12 (StataCorp., Texas, USA). The availability of a wide range of prospectively collected socio-demographic variables in ALSPAC enables to account for confounders and factors that explain missingness, thus supporting the ‘missing-at-random’ assumption. The final imputation model included all study variables and over 60 auxiliary variables relating to family structure, socio-demographic factors, maternal and child characteristics that have been identified as strong predictors of missingness in the confounders and the outcome. To assist with the imputation of the key model variables we included (1) repeated measures of pubertal development (breast and pubic hair development) based on the Tanner staging system (Tanner 1990) as a proxy for missing age at menarche (Joinson et al. 2012); (2) repeated measures of depression in the offspring assessed through the computerised version of the Clinical Interview Schedule-Revised (CIS-R; Lewis et al. 1992) at various measurement occasions; and (3) earlier measures of depressive symptoms as a proxy for missing depressive symptoms. Fifty datasets by 10 cycles of regression were generated. The imputation was restricted to the estimation of the outcome, with an increase in the sample size due to inclusion of Tanner staging data (girls who had either age at menarche or any measure of Tanner stage were included; *n* = 4534). The analyses were repeated by averaging parameter estimates over the imputed datasets, and computing the associated standard errors using Rubin’s rule (Rubin 1987). Standard errors and 95 % confidence intervals for direct and indirect effects in the imputed data analyses were estimated using bootstrapping methodology (1000 samples). Analyses were performed using the sample with complete and imputed data. However, due to non-trivial rates of attrition, final models are based on the analysis with imputed data (results of the complete case analysis are provided in Online Resource 1).

## Results

### Characteristics of the Sample

Analysis of the socio-demographic differences across various samples indicated that the study sample comprised individuals who were more advantaged (i.e., had lower rates of socioeconomic disadvantage) compared to those who were lost to attrition (Table 1). Those with complete data were more likely to come from families of non-manual social class and to have mothers who gave birth at a later age, had fewer children, and were educated beyond high school. The study sample also comprised a higher percentage of girls from families who owned their home, had access to a car and were less likely to experience financial problems. There was evidence that girls with missing data on depressive symptoms were more likely to be from father-absent homes and had earlier menarche (results are provided in Online Resource 2).

### Distribution of Age at Menarche and Depressive Symptoms in Father-Present and Father-Absent Samples, and by Socioeconomic Status and Maternal Characteristics

Girls from father-absent homes had earlier menarche and reported higher levels of depressive symptoms than girls from father-present homes (Table 2). There was evidence that girls from families who resided in rented accommodation and those who experienced financial problems had higher levels of depressive symptoms and earlier menarche (Table 3). Girls whose mothers had antenatal depression reported higher levels of depressive symptoms and earlier menarche than girls whose mothers had not. Similarly, girls whose mothers reported earlier age at menarche had higher levels of depressive symptoms and earlier menarche than those whose mothers experienced menarche at a normative or late age. There was no evidence that girls whose mothers had or had no qualifications differed in terms of reported levels of depressive symptoms. However, girls reported earlier menarche if their mothers had no qualifications.

**Table 2:** Means and standard deviations of the mediator (age at menarche) and outcome (depressive symptoms)^a^ variables in father-present and father-absent samples

Risk factor	Age at menarche (*n* = 3785)			Depressive symptoms (*n* = 2862)		
	Mean	S.D.	*t*-test, *p*	Mean	S.D.	*t*-test, *p*
Father present	12.68	1.14	5.36, <0.001	5.58	4.80	−3.85, <0.001
Father absent (birth to 5 years)	12.39	1.23		6.62	5.42	

^a^Higher scores indicate higher levels of depressive symptoms

**Table 3:** Means and standard deviations for levels of depressive symptoms at 14 years (short mood and feelings questionnaire [SMFQ] ≥11)^a^ and age at menarche by socioeconomic status and maternal characteristics

Risk factors	Depressive symptoms				Age at menarche			
	Sample size (n)	Mean	S.D.	*t*-statistic, *p*	Sample size (n)	Mean	S.D.	*t*-statistic, *p*
Major financial problems								
No	2627	5.55	4.86	3.11, 0.002	3524	12.66	1.16	−3.34, 0.001
Yes		6.51	5.19			12.45	1.18	
Homeownership status								
Owned	2818	5.57	4.81	−2.79, 0.005	3793	12.67	1.14	4.30, ≤0.001
Privately rented		6.26	5.24			12.46	1.25	
Mother’s educational attainment								
≥High school	2817	5.69	4.91	−0.01, 0.997	3775	12.67	1.16	3.87, ≤0.001
No qualifications		5.69	4.89			12.50	1.15	
Maternal age at menarche								
Normative/Late (12–15 years)	2534	5.58	4.80	−2.24, 0.025	3411	12.74	8.67	13.34, ≤0.001
Early (8–11 years)		6.15	5.30			12.09	1.14	
Maternal depression								
No	2632	5.45	4.80	−4.51, <0.001	3505	12.66	1.15	3.02, 0.002
Yes		6.54	5.26			12.51	1.21	

Sample sizes vary because of differences in data availability for indicators of socioeconomic disadvantage and maternal characteristics

^a^Higher scores indicate higher levels of depressive symptoms

### Measurement Model

**Fig. 1:**
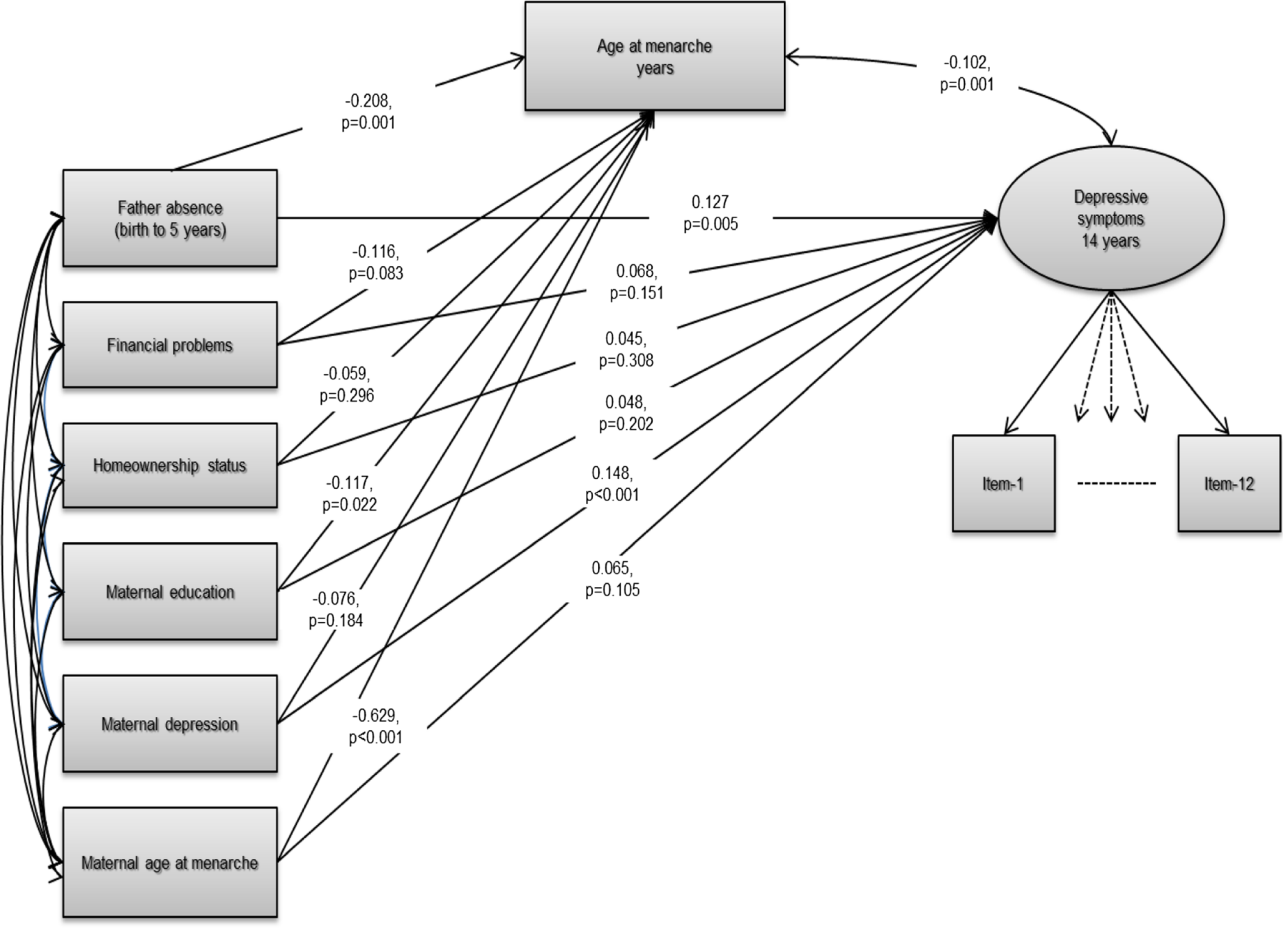
Structural model of the association between early childhood father absence and depressive symptoms, adjusted for antenatal socioeconomic and maternal confounders (*n* = 4534). *Note*. Analysis was performed on the imputed sample

The hypothesised pathways and the role of observed mediator-exposure confounding are illustrated in Fig. 1. The observed variables are displayed in boxes and latent outcome variable as a circle. The overall fit of the models was assessed using the comparative fit index/Tucker-Lewis index (CFI/TLI, >0.9 desirable; Bentler 1990) and the root mean square error of approximation (RMSEA, <0.05 desirable; Steiger 1990). The chi-square test of overall fit is overly sensitive to model specification when samples size is large or the observed variables are non-normally distributed (Kline 2011). Depressive symptoms at 14 years were modelled as a latent trait using a confirmatory factor analysis (CFA) model with a factor being measured by 12 categorical items from the SFMQ (variable loadings on the latent factor are provided in Online Resource 3). Model fit statistics indicated that the model had an acceptable fit (CFI/TLI = 0.98/0.98; RMSEA = 0.05 95 % CI [0.044, 0.050]) suggesting that our construct was adequately measured.

### Mediation Model

Two models were tested to examine the hypothesised mediation effect whilst controlling for the mediator-outcome confounding. First, the unadjusted model that included only the exposure, mediator, and the outcome was estimated. Second, the model adjusted for antenatal indices of socioeconomic disadvantage (financial problems, homeownership status, and maternal educational attainment) and maternal characteristics (mother’s age at menarche and depressive symptoms) was estimated. Table 4 shows path estimates (*β*) of the direct and indirect (mediated through age at menarche) effects in the unadjusted and adjusted models. Within the unadjusted model, there was strong evidence for an indirect effect from early childhood father absence to depressive symptoms at 14 years through age at menarche. There was strong evidence for the direct effect from father absence to depressive symptoms in mid-adolescence once the indirect effect through age at menarche was accounted for.

**Table 4:** Estimates of the direct effect and effect mediated through age at menarche in the association between early childhood father absence and depressive symptoms adjusted for antenatal socioeconomic and maternal confounders in imputed sample (*n* = 4534)

	Age at menarche			Bootstrapping	
Effect size^a^	*β*	SE	Z	BC 95 % CI	*p*
	Unadjusted model				
Father absence on depressive symptoms, unadjusted for age at menarche	0.173	0.042	4.076	0.091, 0.255	≤0.001
Father absence on age at menarche	−0.285	0.057	−5.034	−0.397, −0.173	≤0.001
Father absence on depressive symptoms, adjusted for age at menarche (direct effect)	0.148	0.042	3.512	0.066, 0.230	≤0.001
Father absence on depressive symptoms, through age at menarche (indirect effect)	0.025	0.007	3.705	0.011, 0.039	≤0.001
	Adjusted model^b^				
Father absence on depressive symptoms, unadjusted for age at menarche	0.127	0.045	2.832	0.039, 0.215	0.005
Father absence on age at menarche	−0.208	0.060	−3.471	−0.326, −0.090	0.001
Father absence on depressive symptoms, adjusted for age at menarche (direct effect)	0.110	0.044	2.475	0.024, 0.196	0.013
Father absence on depressive symptoms, through age at menarche (indirect effect)	0.017	0.006	2.792	0.005, 0.029	0.005

*BC* bias corrected (1000 bootstrap samples)

^a^ *Effect Size*: Unadjusted and adjusted regression coefficients; *β*

^b^ *Adjusted model*: Adjusted for indices of socioeconomic background (home ownership status, major financial problems, mother’s educational attainment) and maternal characteristics (early parenthood, maternal antenatal depression, mother’s age at menarche)

Adjustment for antenatal indices of socioeconomic disadvantage and maternal characteristics made little difference to the parameter estimates. Within the fully adjusted model, there remained evidence for an indirect effect from early childhood father absence through age at menarche to depressive symptoms in mid-adolescence (*β* = 0.017, 95 % CI [0.005, 0.029], *p* = 0.005). The indirect effect through age at menarche accounted for approximately 15 % of the total estimated association between father absence from birth to 5 years and depressive symptoms at 14 years. Although attenuated, there was evidence for the direct effect of father absence on depressive symptoms adjusting for age at menarche in the model that controlled for antenatal socioeconomic and maternal confounders. Father absence was strongly associated with earlier menarche in the unadjusted and adjusted models.

Path estimates for the fully adjusted mediation model are illustrated in Fig. 1. Maternal antenatal depression was strongly associated with daughter’s depressive symptoms at 14 years (*β* = 0.148, 95 % CI [0.068, 0.228], *p* < 0.001). Maternal lower educational attainment and earlier age at menarche were associated with earlier menarche in daughters (*β* = −0.117, 95 % CI [−0.217, −0.017], *p* = 0.022 and *β* = −0.629, 95 % CI [−0.735, −0.523], *p* < 0.001, respectively). There was weak evidence for the association between experiences of financial problems in the family and earlier menarche (*β* = 0.116, 95 % CI [−0.247, 0.015], *p* = 0.083). The direction of these associations indicated that girls whose mothers were depressed during the antenatal period were more likely to develop depressive symptoms in mid-adolescence. They were also more likely to commence menses earlier if their mothers reported early age at menarche, had lower educational attainment, and experienced financial problems.

When the analysis was repeated using the sample with complete data, estimates of the direct and indirect effects were in the same direction as they had been in the imputed data analysis and led to the same overarching conclusions. The substantially reduced sample size, however, led to insufficient statistical power to detect these effects. Conducting the analysis on the imputed data addressed the bias due to missing data and improved efficiency of the analysis compared to complete case analysis. Although it is not possible to fully explain the role of response attrition, the pattern of missing data and imputed sample analysis suggested that attrition has led to an underestimation of the effects’ size in the complete case analysis. Here, in estimating the direct and indirect effects, as well as other associations in the model, the standard errors in the imputed sample analysis were smaller than those in the complete case analysis. The improvement in the efficacy may be attributed to the substantial increase in the sample size relative to the complete case analysis, particularly in estimating the direct effect of early childhood father absence on depressive symptoms.

## Discussion

To our knowledge, no previous studies have examined timing of menarche as a mechanism of the association between early childhood father absence and depressive symptoms in adolescence. The findings of this study suggest that early menarche is one pathway through which early childhood father absence is linked to increased levels of depressive symptoms in adolescence. The analysis used SEM to examine mediational pathway whilst controlling for potential mediator–outcome confounding. Approximately 15 % of the total association between early childhood father absence and depressive symptoms was explained by early menarche whilst controlling for various indices of socioeconomic disadvantage and maternal characteristics. Our finding that age at menarche mediated relatively large direct effect may be of conceptual and practical importance even though the size of the indirect effect was relatively small (Preacher and Kelley 2011). In addition to the mediated effect, early childhood father absence was linked to an 11 % increase in depressive symptoms in adolescence from the population mean.

The current study has several strengths, including a large community-based sample, longitudinal design, repeated measures of self-reported depressive symptoms, age at menarche and father absence, and data on a wide range of potential confounders. The robustness of our findings was estimated in the analysis that controlled for possible mediator–outcome confounding. It has been argued that identifying and controlling for such variables should be the focus of mediational research (Cole and Hernan 2002).

A limitation of this study relates to sample attrition which resulted in a relatively high rate of missingness in the outcome and the confounders. Complete case analysis was performed on a relatively small subset (*n* = 2057) comprising individuals with complete data on exposure, outcome, mediator and all confounders. The validity of results based on this analysis depends on the degree to which the data is missing completely at random (MCAR; Schafer and Graham 2002). However, in this study, the missing data mechanism is likely to be that of MAR (Sterne et al. 2009), because girls from lower socioeconomic background with absent fathers and earlier menarche were more likely to have missing data on depressive symptoms (results are provided in Online Resource 2). Although complete case analysis is valid under MCAR assumption, it is inefficient and biased under the MAR, with multiple imputation correcting the later bias (White and Carlin 2010). Inclusion of auxiliary variables that predicted missingness in the outcome and confounders substantiated the MAR assumption, and resulted in a sample of 4534 (>50 % of the total ALSPAC sample of girls) compared to 2057 (>22 %) with complete measures. It should be noted that we cannot rule the possibility that depressive symptoms may be Not Missing At Random (NMAR), and the analytic methods that can address this type of missing data remain underdeveloped (Heron et al. 2011).

Although the study controlled for a range of prospectively measured socioeconomic and maternal confounders, examination of possible genetic confounding factors (e.g., epigenetic changes, interactions with genetic vulnerabilities) that may explain the association between father absence and daughter’s early age at menarche and depressive symptoms was beyond the scope of this study. Results from future genetically informative designs may contribute towards further understanding of the mechanisms underlying the processes involved in biological father absence, earlier age at menarche and depressive symptoms in adolescence. It should also be noted that it is still unclear from the current results whether father absence in early childhood is associated with persistent negative impact on emotional development in girls beyond the mid-adolescence. Further longitudinal studies are needed to examine whether father absence has a long-term impact on rates of depression in late adolescence and early adulthood.

Dysregulation of hypothalamic-pituitary-adrenal (HPA) axis may be an underlying neuroendocrine mechanism linking early childhood father absence, early menarche and depressive symptoms. Early life stress, including that associated with paternal absence and lack of involvement, has been linked to timing of pubertal maturation via heightened activation of HPA axis and long-term alteration of the reproductive hormone axes (Young and Korszun 2010). This finding is in line with theoretical postulations outlined by psychosocial acceleration theory suggesting accelerating effects of psychosocial stress, including absence of the biological father, on timing of pubertal maturation (Belsky et al. 1991). It is possible, therefore, that puberty constitutes a sensitive period for reorganising HPA axis in relation to early life stressors (Quevedo et al. 2012). In line with this argument, recent research indicates that HPA axis activation increases with age, particularly during and after the pubertal transition (Gunnar et al. 2009; Hankin et al. 2010). Hankin et al. (2010) suggest that there is a developmental shift in the pattern of HPA axis activity, with increases in activation among adolescents compared to children. This normative HPA axis increase among healthy youth may be further exacerbated among those at-risk for depressive symptoms.

Similarly, neuroendocrine changes following exposure to early-life stress are increasingly linked with vulnerability to depression in adolescence (Goodyer et al. 2001), particularly in girls who appear to have greater susceptibility to stress-induced HPA axis dysregulation (Heim et al. 2008). The onset of menarche may also trigger latent biological vulnerability to psychosocial, environmental and physiological risk factors increasing the likelihood of psychopathology (Steiner et al. 2003). Thus, dysregulation of normative stress responses induced by experiences of early childhood father absence and associated stresses may render adolescent girls more vulnerable to accelerated menarche and depressive symptoms. However, it is still unclear how neuroendocrine changes associated with exposure to early-life stress and early menarche combine to result in depressive symptoms.

Other associations of interest should be noted. Consistent with previous research, maternal antenatal depression emerged as a strong predictor of depressive symptoms in mid-adolescence (Brand and Brennan 2009). Maternal antenatal depression may be a proxy measure for possible genetic correlates of depressive symptoms that we accounted for in our analysis. Numerous studies suggest a genetic link between maternal depression and depressive symptoms in children and adolescents (Goodman and Gotlib 1999). Similarly, maternal age at menarche may be a proxy measure of genetically transmitted pubertal timing (Thomis and Towne 2006), thus, a strong confounder of the association between daughter’s age at menarche and depressive symptoms. Despite these strong confounders, there was still evidence for the mediating effect of early menarche in the association between early childhood father absence and depressive symptoms in adolescent girls. In line with previous research, early childhood father absence was strongly associated with early menarche even after controlling for a range of socioeconomic and maternal confounders (Ellis 2004). Although father absence is not an isolated event, but a process which begins before the father departs, controlling for other adversities in child’s environment did not account for the negative effect of father absence on adolescent girls’ mental health. Once these factors were accounted for, father absence still explained variability in depressive symptoms.

The current findings have important implications for understanding the aetiology of depressive symptoms. Young girls at risk for depressive symptoms as a function of stressful family factors (e.g., biological father absence) and earlier menarche may benefit from early preventive strategies. Early prevention efforts may be of particular importance considering possible hormonal and neuroendocrine vulnerability that is triggered by exposure to father absence and associated stress. Father absence and age at menarche are factors that cannot be directly targeted by intervention programs. However, interventions focusing on promoting problem solving and social skills, as well as positive cognitions (e.g., self-esteem) may prove effective.

## Electronic supplementary material

Below is the link to the electronic supplementary material.

Online Resource 1 (DOC 40 kb)

Online Resource 2 (DOC 40 kb)

Online Resource 3 (DOC 39 kb)
