# Supplementary material for: Early Childhood Father Absence and Depressive Symptoms in Adolescent Girls from a UK Cohort: The Mediating Role of Early Menarche
Source: J Abnorm Child Psychol. 2014 Nov 20;43(5):921–31. doi: 10.1007/s10802-014-9960-z (PMC4465664; doi:10.1007/s10802-014-9960-z)
Supplement: Supplementary file 1 — (DOC 40 kb) [file 10802_2014_9960_MOESM1_ESM.doc]

**Online Resource 1**

**Journal of Abnormal Child Psychology**

**Early Childhood Father Absence and Depressive Symptoms in Adolescent Girls from a UK Cohort: the Mediating Role of Early Menarche**

Iryna Culpin PhD^1^, Jon Heron PhD^1^, Ricardo Araya PhD MRCPsych^1^, Carol Joinson PhD^1^

^1^ School of Social and Community Medicine, University of Bristol, UK

Corresponding author: Iryna Culpin; Email: [Iryna.Culpin@bristol.ac.uk](mailto:Iryna.Culpin@bristol.ac.uk)

**Table**  Estimates of the Direct Effect and Effect Mediated through Age at Menarche in the Association between Early Childhood Father Absence and Depressive Symptoms Adjusted for Antenatal Socioeconomic and Maternal Confounders in Complete Case Sample (n=2,057)

|  | Age at menarche | | | Bootstrapping | |
| --- | --- | --- | --- | --- | --- |
| Effect Size ^a^ | Unadjusted model | | |  | |
|  | *β* | SE | Z | BC 95% CI | *p* |
| Father absence on depressive symptoms, unadjusted for age at menarche | 0.190 | 0.081 | 2.360 | 0.062, 0.330 | 0.019 |
| Father absence on age at menarche | -0.219 | 0.085 | -2.566 | -0.359, -0.076 | 0.010 |
| Father absence on depressive symptoms, adjusted for age at menarche (direct effect) | 0.169 | 0.080 | 2.119 | 0.042, 0.307 | 0.034 |
| Father absence on depressive symptoms, through age at menarche (indirect effect) | 0.021 | 0.009 | 2.239 | 0.008, 0.040 | 0.025 |
|  | Adjusted model ^b^ | | | | |
| Father absence on depressive symptoms, unadjusted for age at menarche | 0.108 | 0.086 | 1.259 | -0.034, 0.277 | 0.208 |
| Father absence on age at menarche | -0.165 | 0.085 | -1.948 | -0.315, -0.034 | 0.051 |
| Father absence on depressive symptoms, adjusted for age at menarche (direct effect) | 0.093 | 0.085 | 1.094 | -0.050, 0.235 | 0.274 |
| Father absence on depressive symptoms, through age at menarche (indirect effect) | 0.015 | 0.009 | 1.749 | 0.004, 0.033 | 0.080 |

*^a^ Effect Size*: Unadjusted and adjusted regression coefficients; *β*

*^b^ Adjusted model*: Adjusted for indices of socioeconomic background (home ownership status, major financial problems, mother's educational attainment) and maternal characteristics (early parenthood, maternal antenatal depression, mother's age at menarche)

*Note*. BC: bias corrected (1,000 bootstrap samples)
