# Supplementary material for: Early Childhood Father Absence and Depressive Symptoms in Adolescent Girls from a UK Cohort: The Mediating Role of Early Menarche
Source: J Abnorm Child Psychol. 2014 Nov 20;43(5):921–31. doi: 10.1007/s10802-014-9960-z (PMC4465664; doi:10.1007/s10802-014-9960-z)
Supplement: Supplementary file 2 — (DOC 40 kb) [file 10802_2014_9960_MOESM2_ESM.doc]

**Online Resource 2**

**Journal of Abnormal Child Psychology**

**Early Childhood Father Absence and Depressive Symptoms in Adolescent Girls from a UK Cohort: the Mediating Role of Early Menarche**

Iryna Culpin PhD^1^, Jon Heron PhD^1^, Ricardo Araya PhD MRCPsych^1^, Carol Joinson PhD^1^

^1^ School of Social and Community Medicine, University of Bristol, UK

Corresponding author: Iryna Culpin; Email: [Iryna.Culpin@bristol.ac.uk](mailto:Iryna.Culpin@bristol.ac.uk)

**Table** Predictors of Missingness in the Outcome, Exposure and Mediator in the ALSPAC Cohort of Adolescent Girls (n=7,056)

|  | Odds of missingness in key variables | | | | | |
| --- | --- | --- | --- | --- | --- | --- |
|  | Depressive symptoms ^a^ | | Father absence (birth to 5 years) | | Age at menarche ^b^ | |
|  | OR | 95% CI, *p* | OR | 95% CI, *p* | OR | 95% CI, *p* |
| Depressive symptoms 14y ^a^ (n=3,073) | _ | _ | 1.11 | 0.77 – 1.62, 0.569 | 0.87 | 0.59 – 1.27, 0.472 |
| Father absence 0-5 y (n=5,295) | 2.11 | 1.83 – 2.44, <0.001 | _ | _ | 2.17 | 1.88 – 2.51, <0.001 |
| Age at menarche ^b^ (n=4,148) | 1.47 | 1.32 – 1.64, <0.001 | 1.24 | 1.05 – 1.48, 0.014 | _ | _ |
| Major financial problems (n=5,790) | 0.64 | 0.55 – 0.75, <0.001 | 0.66 | 0.55 – 0.78, <0.001 | 0.70 | 0.60 – 0.81, <0.001 |
| Homeownership status (n=6,344) | 2.01 | 1.82 – 2.21, <0.001 | 1.87 | 1.70 – 2.06, <0.001 | 2.01 | 1.83 – 2.20, <0.001 |
| Mother’s educational attainment (n=6,031) | 2.44 | 2.17 – 2.74, <0.001 | 2.66 | 2.33 – 3.05, <0.001 | 2.24 | 1.99 – 2.50, <0.001 |
| Maternal antenatal depression (n=5,596) | 1.56 | 1.38 – 1.77, <0.001 | 2.04 | 1.76 – 2.37, <0.001 | 1.52 | 1.34 – 1.73, <0.001 |
| Maternal age at menarche (n=5,440) | 1.23 | 1.08 – 1.41, <0.001 | 1.25 | 1.06 – 1.48, 0.009 | 1.11 | 0.97 – 1.27, 0.135 |

^a^ Binary measure of depressive symptoms was derived using the cut-off point of 11 (≥11=high levels of depressive symptoms)

^b^ Binary measure of age at menarche was derived summarising timing of menarche as normative/late versus early (11.6≤12.6≥13.6)
