# Supplementary material for: Early Childhood Father Absence and Depressive Symptoms in Adolescent Girls from a UK Cohort: The Mediating Role of Early Menarche
Source: J Abnorm Child Psychol. 2014 Nov 20;43(5):921–31. doi: 10.1007/s10802-014-9960-z (PMC4465664; doi:10.1007/s10802-014-9960-z)
Supplement: Supplementary file 3 — (DOC 39 kb) [file 10802_2014_9960_MOESM3_ESM.doc]

**Online Resource 3**

**Journal of Abnormal Child Psychology**

**Early Childhood Father Absence and Depressive Symptoms in Adolescent Girls from a UK Cohort: the Mediating Role of Early Menarche**

Iryna Culpin PhD^1^, Jon Heron PhD^1^, Ricardo Araya PhD MRCPsych^1^, Carol Joinson PhD^1^

^1^ School of Social and Community Medicine, University of Bristol, UK

Corresponding author: Iryna Culpin; Email: [Iryna.Culpin@bristol.ac.uk](mailto:Iryna.Culpin@bristol.ac.uk)

**Table** Factor Loadings and Fit Indices for the Measurement Model

| Item | Factor loading | S.E. |
| --- | --- | --- |
| I felt miserable or unhappy | 0.746 | 0.012 |
| I didn’t enjoy anything at all | 0.555 | 0.016 |
| I felt so tired I just sat around and did nothing | 0.414 | 0.016 |
| I felt I was no good any more | 0.883 | 0.008 |
| I cried a lot | 0.729 | 0.012 |
| I found it hard to think properly or concentrate | 0.598 | 0.014 |
| I hated myself | 0.899 | 0.008 |
| I was a bad person | 0.690 | 0.014 |
| I felt lonely | 0.813 | 0.009 |
| I though nobody really loved me | 0.860 | 0.009 |
| I thought I could never be as good as other kids | 0.802 | 0.010 |
| I did everything wrong | 0.822 | 0.010 |
|  |  |  |
| Free parameters | 24 |  |
| Comparative fit index | 0.984 |  |
| Tucker-Lewis index | 0.981 |  |
| Root mean square error of approximation | 0.047 (95% CI  [0.044, 0.050]) |  |

The Table shows factor loadings and fit indices for the measurement model. The measurement model was derived using as much data as possible, disregarding the availability of other covariates. Item ‘I was very restless’ was omitted from the 13-item SMFQ in this analysis because previous work in ALSPAC indicated that some individuals in the study sample were uncertain about the meaning of this item. It is clear from the table that although all items loaded significantly on the depressive symptoms latent trait, there was a wide variability in the estimated loadings. For instance, item ‘I felt so tired I just sat around and did nothing’ (0.414) is relatively unimportant compared with ‘I hated myself’ (0.899), ‘I felt I was no good any more’ (0.883) and ‘I though nobody really loved me’ (0.860). Given these data, it is evident that the assumption of equal loadings underlying the simple sum-score would miss the large discrepancy between the items. Inspection of model fit statistics indicated that the model had an acceptable fit (CFI/TLI=0.984/0.981; RMSEA=0.05 95% CI [0.044, 0.050]) suggesting that the construct (depressive symptoms latent trait) was adequately measured. This supported the adequacy of the model for subsequent tests of structural paths and mediation effect.
